# Barrier-Restoring Therapies in Atopic Dermatitis: Current Approaches and Future Perspectives

**DOI:** 10.1155/2012/923134

**Published:** 2012-08-22

**Authors:** Y. Valdman-Grinshpoun, D. Ben-Amitai, A. Zvulunov

**Affiliations:** ^1^Pediatric Dermatology Unit, Schneider Children's Medical Center of Israel, 49202 Petach Tikva, Israel; ^2^Department of Dermatology, Szold Health Center, Clalit Health Services, 84894 Beer-Sheva, Israel; ^3^Sackler Faculty of Medicine, Tel Aviv University, 69978 Tel Aviv, Israel; ^4^Medical School for International Health, Faculty of Medicine, Ben-Gurion University of the Negev, 84105 Beer-Sheva, Israel

## Abstract

Atopic dermatitis is a multifactorial, chronic relapsing, inflammatory disease, characterized by xerosis, eczematous lesions, and pruritus. The latter usually leads to an “itch-scratch” cycle that may compromise the epidermal barrier. Skin barrier abnormalities in atopic dermatitis may result from mutations in the gene encoding for filaggrin, which plays an important role in the formation of cornified cytosol. Barrier abnormalities render the skin more permeable to irritants, allergens, and microorganisms. Treatment of atopic dermatitis must be directed to control the itching, suppress the inflammation, and restore the skin barrier. Emollients, both creams and ointments, improve the barrier function of stratum corneum by providing it with water and lipids. Studies on atopic dermatitis and barrier repair treatment show that adequate lipid replacement therapy reduces the inflammation and restores epidermal function. Efforts directed to develop immunomodulators that interfere with cytokine-induced skin barrier dysfunction, provide a promising strategy for treatment of atopic dermatitis. Moreover, an impressive proliferation of more than 80 clinical studies focusing on topical treatments in atopic dermatitis led to growing expectations for better therapies.

## 1. Introduction

Atopic dermatitis (AD) is a multifactorial, chronic relapsing, inflammatory disease, characterized by xerosis, eczematous lesions, and pruritus. AD usually begins in infancy or early childhood, about 90% of cases start in first five years of life [1]. The disease has significant morbidity and it adversely affects the quality-of-life of the child and his family in both social and emotional aspects [2]. The most dominant physical symptoms experienced by affected children are sleep disturbances and pruritus/scratching. It is important to notice that pruritus usually leads to an “itch-scratch” cycle that may compromise the epidermal barrier, resulting in transepidermal water loss (TEWL), xerosis, or secondary infection, especially with *Staphylococcus aureus *[3].

There are many theories regarding the pathogenesis of AD. The pathogenesis of AD involves skin barrier dysfunction, environmental and infectious agents, and immune abnormalities. In 1999 Elias and Taieb proposed that failure of the skin barrier may be the primary factor in the development of AD [4, 5]. Subsequent studies demonstrated epidermal barrier abnormalities in AD dysfunction that correlate with the disease severity. TEWL is greater in areas with clinical disease. Even clinically uninvolved sites of skin show abnormal skin barrier function and greater TEWL compared to healthy individuals [6–9]. However, it must be noted that TEWL is only relevant when regarding the penetration of molecules less than 500 daltons, such as water, irritants, and haptens.

## 2. Skin Barrier Dysfunction in AD

Skin is a barrier that protects the body from the outside world. Defense functions are localized in the stratum corneum (SC), which typically includes about 9–15 corneocyte cell layers that consist of packing of keratin filaments and filaggrin of corneodesmosomes [10]. Elias depicted the SC as a brick wall, with the corneocytes analogous to bricks and lipid lamellae acting as mortar [11]. These lipids are composed of approximately 50% ceramide, 25% cholesterol, and some long-chain free fatty acid. Lipid lamellae play a crucial role in the barrier function [12]. Sphingosine, ceramide metabolite, exhibits potent *in vitro* antimicrobial activity [13, 14], and is reduced in AD patient's skin [15–17], predisposing to pathogen colonization. 

The pathogenesis of AD is not completely understood. Nevertheless, congenital and acquired defects in each part of the SC structure are associated with pathogenesis of AD. Furthermore, skin barrier abnormalities in AD may result from mutations in the gene encoding for filaggrin, which plays an important role in the formation of cornified cytosol. The products of filaggrin breakdown are important for hydration and acidification of the SC, which are both impaired in AD [9]. Abnormal maturation and secretion of lamellar body in AD results in reduction of lipids and ceramides content and increased cholesterol levels in AD as compared to nonatopic subjects [18, 19]. These barrier abnormalities render the skin more permeable to irritants, allergens, and microorganisms [20]. Conversely, pathogen colonization further impairs the abnormality of the permeability barrier [21]. *Staphylococcus aureus* colonization on the skin may be found in up-to 90% of AD patients [22]. Moreover, *Staphylococcus aureus* may produce ceramidase, which additionally undermines the barrier function [15]. 

Severe pruritus is the most disturbing symptom of AD. The scratching severely compromises the skin barrier, enhancing inflammatory reacting that subsequently results in the vicious itch-scratch cycle.

## 3. Therapeutic Aspects

Treatment of AD must be directed to control the itching, suppress the inflammation, and restore the skin barrier. There are various strategies and medical efforts that can help us achieve these goals. In addition, it is extremely important to educate the parents, emphasizing the chronic nature of the disease, the importance of continued maintenance therapy, and the need for prompt suppression of flare-ups. Patients should also be provided with written instructions regarding appropriate medical care in order to reinforce learning [20, 23–25]. 

## 4. Emollients

Emollients are widely used in conservative local treatment of AD. There are few objective studies based on clinical evidence demonstrating their efficiency [26–28]. Emollients, both creams and ointments, improve the barrier function of SC by providing it with water and lipids. Nevertheless, the exact mechanism by which this process works is still unknown [29]. Ghadially et al. showed that petrolatum lipids may replace SC bilayers and accelerate barrier recovery in human skin [30]. 

A case-control study by Macharia et al. demonstrated that the use of topical petrolatum in infants may protect against AD development [31]. Additionally, several studies have demonstrated a reduction in incidence of “dermatitis” or improved skin condition in premature neonates treated with emollients [32–36]. One recent pilot study on primary prevention of AD by emollients therapy starting in infancy has shown some promising results [37]. 

Emollients have been shown to enhance the effects of topical corticosteroids (TCS) therapy in children with AD in a randomized comparison study [38] and lead to reduced usage of TCS [39].

Though usually effective in a short range, most of emollient moisturizers contain nonphysiologic lipids, such as petrolatum, lanolin mineral oil, and silicone. These substances may impede, rather than correct, the underlying biochemical response of the skin barrier's flawed structure in AD [40]. A 1996 study proclaimed that application of petrolatum in damaged skin results in a partially restored barrier function in acute injury models, but this benefit is fairly short [41].

## 5. Barrier Repair Therapy

Recent advances in the understanding of pathophysiology of the epidermal barrier and its critical role in the pathogenesis of AD led to increased interest in barrier repair therapies. But what does “barrier repair therapy” mean? Ideally, the emollients should normalize the epidermal barrier function by reducing TEWL and improving SC hydration [42]. 

Properties of physiologic lipid-based products are different from nonphysiologic agents. Lipids are taken up by keratinocytes, packaged into the lamellar bodies, and then re-secreted to form lamellar bilayers. Equimolar ratio of 1 : 1 : 1 of ceramide, cholesterol, and FFA induces barrier recovery in acute injure models [41]. 

Studies on AD and barrier repair treatment, either in animal models or in humans, showed that adequate lipid replacement therapy reduces the inflammation and restores epidermal function comparable to topical fluticasone cream [23, 43–47]. 

Urea, a well-known humectant used in various topical emollients, has been very recently shown to normalize barrier function and antimicrobial peptide (LL-37 and *β*-defensin-2) expression in a murine model of AD [48].

There are several nonprescription products that proclaim barrier repair properties [42, 49]. Chinese herbal mixtures (CHM) had been often claimed beneficial in treatment of AD. A recent study had demonstrated that topical CHM accelerated barrier recovery following acute barrier disruption by increased epidermal lipid content and mRNA expression of fatty acid and ceramide synthetic enzymes, mRNA levels for the epidermal glucosylceramide transport protein, and mRNA expression of antimicrobial peptides both *in vivo* and *in vitro* [50].

Skin care products that contain high lipid substances are frequently applied for the care of dry skin and inflammatory skin conditions [51]. Oils, both pure and integrated, are commonly applied for skin care. The oils assist the native lipids of the SC to provide a better barrier function and consequently help moisturizing the skin [52]. The decreased TEWL values specify that the use of the oils leads to a semiocclusion of the skin surface. Similar results were attained for both mineral and vegetable oils [53]. Paraffin, jojoba, and almond oils were shown to penetrate equally into layers of SC [52], while coconut oil, used as a moisturizer, was found to be as effective and safe as paraffin oil [54]. Proksch et al. found that bathing in magnesium-rich (5%) Dead Sea salt solution improves skin barrier function, augments skin hydration, and decreases inflammation in atopic dry skin [55]. A recent study revealed that treatment of atopic dermatitis by a Dead Sea mineral enriched body cream, improves physiologic and clinical severity scores of the disease, and may serve as a maintenance therapy for AD patients [56]. 

## 6. Topical Corticosteroids and**** Immunomodulators

The inflammatory response of AD is mediated by lymphocytes, mast cells, eosinophil, dendritic cells, and monocytes/macrophages [23, 57]. TCS may inhibit many aspects of inflammation in AD, thus it is still being used as a standard therapy, especially for acute flare-ups. The effects of TCS are facilitated by cytoplasmic glucocorticoids receptors in various types of cells, such as keratinocytes and fibroblasts, as well as in immune cells. When activation occurs, the receptor binds to glucocorticoids response elements in the promoter region of target genes. Consequently, the receptor inhibits the transactivating function of transcription factors, which results in reduced expression of proinflammatory genes [58]. 

Recently, nerve growth factor (NGF), substance P, and eosinophil count, were all found elevated in the plasma of AD patients. These are considered possible mechanisms of itch in AD [59, 60]. The treatment of AD with TCS results in NGF reduction and in relief of pruritus [59]. 

Tacrolimus and Pimecrolimus are topical calcineurin inhibitors (TCI). These steroidal-free alternatives in the treatment of the inflammatory response in AD constitute the second line therapy in AD [61]. The action mechanism of TCI is limited to immune cells only, thus skin atrophy or telangiectasia are not observed, contrary to TCS [62]. Consequently, we may use TCI in sensitive affected area, such as face, eyes, neck, and genitalia, without concern of systemic absorption or skin atrophy [3, 24]. 

Although effectively reducing inflammation by suppressing immune reaction in AD, TCS, and TCI do not correct the primary skin barrier abnormality that principal the pathogenesis of the disease [44]. Recent studies have shown that the use of TCI or TCS may compromise skin barrier function in normal skin [25, 63, 64]. 

One recent study demonstrates that betamethasone and pimecrolimus improve clinical and biophysical parameters of barrier function, but differ in their effects on the epidermal barrier. 

Betamethasone employed a more effective antiproliferative and anti-inflammatory result, leading to a faster reduction in TEWL, but causing epidermal thinning. 

Pimecrolimus indicating renovation of the epidermal barrier by inducing regular lipid layer formation and lamellar body extrusion. 

## 7. Treatment of Skin Infections

Following the compromising of antimicrobial barrier in AD, there is colonization of *Staphylococcus aureus* in AD patients, even in nonlesional skin [22]. Furthermore, superantigen produced by *Staphylococcus aureus* strains colonize more commonly in steroid-resistant patients [65]. 

Recent findings proclaim that barrier skin permeability and antimicrobial function share common structural and biochemical features, and both are coregulated and interdependent [16, 66]. As a consequence, secondary infections may be triggered by failure of the permeability barrier. Contrariwise, pathogen colonization or infection may exacerbate the permeability barrier abnormality [21]. 

Presence of secondary bacterial infection in AD lesions may require a short-term topical or systemic antibiotic therapy. Conversely, some researchers claim that barrier repair therapy may reduce secondary colonization of pathogenic *Staphylococcus aureus* by targeted correction of lipid biochemical abnormalities [21].

## 8. Future Perspectives

Inflammation itself may be able to induce a functional dysfunction and induce or aggravate AD [67]. Consequently, efforts directed to develop immunomodulators that interfere with cytokine-induced skin barrier dysfunction, provide a promising “kill two birds with one stone” strategy for treatment of AD.

Potential therapeutic use of phosphodiesterase-4 inhibitors in a variety of inflammatory disease, including AD, has been known for years. However so far, an appropriate molecule devoid of gastrointestinal adverse effects has not been approved. Currently, there are several studies exploring various topically administered PDE4 inhibitors that suppress the release of TNF-*α*, IL-12, IL-23, and other cytokines [68].

Bissonnette et al. recently reported beneficial clinical effects in adults with AD [69]. This compound is a novel small molecule, derived from metabolites of a unique group of bacterial symbionts (organisms in a symbiotic relationship; the symbiont is the smaller of these and is always a beneficiary in the relationship, while the larger organism is the host and may or may not derive a benefit) of entomopathogenic nematode that has been shown to inhibit inflammatory cytokine secretion by activated T cells, including tumor necrosis factor-*α* and interferon-*χ* *in vitro*.

Fucoidan, a sulphated polysaccharide extracted from brown seaweed having a wide range of pharmacological, has been very recently shown to significantly inhibit mRNA expression of TARC, MDC, and RANTES chemokines and improve clinical features of AD in mice model comparable to topical dexamethazone 0.1% [70].

Finally, an impressive proliferation of more than 80 clinical studies focusing on topical treatments in atopic dermatitis, many of these involving new or novel active ingredients (http://www.clinicaltrials.gov), led to growing expectations for better therapies. These include DPK-060 by DermaGen-AB, BPR277 by Novartis, GW842470X by GlaxoSmithKline, KP-413 by Kaken Pharmaceutical, TS-022 by Taisho Pharmaceutical R&D Inc., LAS41002 by Almirall, S.A., CD2027 by Galderma, BRT-FC-83C by Biomed Research & Technologies, Inc., E6005 by Eisai Co., Ltd., PH-10 by Provectus Pharmaceuticals, V0034CR01B by Pierre Fabre, Mapracorat and ZK245186 by Intendis GmbH, SRD174 by Serentis Ltd., and 0416 and 0417 by Fougera Pharmaceuticals Inc. Unfortunately, for most of these substances, nature and properties are still kept confidential.
